# Taxonomy through the lens of neutral helium microscopy

**DOI:** 10.1038/s41598-018-36373-5

**Published:** 2019-02-14

**Authors:** Thomas A. Myles, Sabrina D. Eder, Matthew G. Barr, Adam Fahy, Joel Martens, Paul C. Dastoor

**Affiliations:** 1 Centre for Organic Electronics, University of Newcastle, Callaghan, New South Wales, 2308 Australia; 2 Department of Physics and Technology, University of Bergen, Allégaten 55, 5007 Bergen, Norway

## Abstract

The field of taxonomy is critically important for the identification, conservation, and ecology of biological species. Modern taxonomists increasingly need to employ advanced imaging techniques to classify organisms according to their observed morphological features. Moreover, the generation of three-dimensional datasets is of growing interest; moving beyond qualitative analysis to true quantitative classification. Unfortunately, biological samples are highly vulnerable to degradation under the energetic probes often used to generate these datasets. Neutral atom beam microscopes avoid such damage due to the gentle nature of their low energy probe, but to date have not been capable of producing three-dimensional data. Here we demonstrate a means to recover the height information for samples imaged in the scanning helium microscope (SHeM) via the process of stereophotogrammetry. The extended capabilities, namely sparse three-dimensional reconstructions of features, were showcased via taxonomic studies of both flora (*Arabidopsis thaliana*) and fauna (*Heterodontus portusjacksoni*). In concert with the delicate nature of neutral helium atom beam microscopy, the stereophotogrammetry technique provides the means to derive comprehensive taxonomical data without the risk of sample degradation due to the imaging process.

## Introduction

Taxonomy, the science and practice of classification based on shared characteristics^1^, is a crucial tool for the identification of species^2^ as well as their conservation and ecology^3–6^. Indeed, accurate and replicable taxonomic identification has been described as the cornerstone of biology; a means to prevent research from becoming irreproducible and thus of reduced value to the scientific community^7^. With only 1.5 million of an estimated 5 ± 3 million extant species described sufficiently, the field is actively searching for new technologies to improve methodology and hence process an ever broader array of species^8–10^. Morphology-based taxonomy often relies on microscopy techniques to identify features too small to be resolved by the human eye, and in recent years the field has moved towards incorporating three-dimensional imaging techniques to offer additional description methods for taxonomic applications^10–12^. Whilst these imaging techniques, such as the scanning electron microscope (SEM)^12,13^, X-ray microtomography (microCT)^10^, and confocal microscopy^14^, have greatly benefited the field, there still remains a number of challenges due to the delicate nature of a wide range of organisms and structures. Damage to the sample under investigation is of concern during the imaging process or resulting from sample preparation. Radiation damage, photobleaching, sputtering, and anti-charging surface coatings can all lead to degradation of fragile biological samples; potentially altering the size, shape, and chemistry of features of interest^15^. Any such morphological changes could call into question the validity of the produced experimental results.

The atom-surface interaction of a ground-state neutral helium atom has long been exploited by the field of helium atom scattering (HAS) as a completely non-destructive means of probing a sample surface^16^. Milli-electron volt beam energies (orders of magnitude lower than comparable beams of electrons or photons), in tandem with the inherent properties of the helium atom – electrically neutral, chemically inert, and a lack of interaction with electric and magnetic fields - yields an ideal probe for delicate material systems. Furthermore, these same properties ensure unambiguous surface sensitivity (no penetration into the bulk) and no surface charging, thus removing the need for intrusive sample preparations. The theoretical resolution of a thermal neutral helium probe is limited only by the intrinsic wavelength (~0.5 Å) of the helium atoms^17^. The field of neutral helium atom microscopy^18–21^ utilises these properties to permit imaging of a wide range of samples, including those that are susceptible to damage.

The scanning helium microscope (SHeM) is a pinhole-based, neutral helium microscope capable of producing micrographs with image contrast originating not only from topographic features^22^, but also from the local chemical composition^23^. The produced micrographs are intuitive and give the impression of depth; in part due to the instrument’s large depth-of-field^24^. Similar to other scanned beam techniques, SHeM micrographs are projections of three-dimensional surface structures onto two-dimensional planes and thus direct information concerning height is lost^25^. Accurate taxonomy relies on the specifics of the shape, texture and aspect ratio of microscopic structures. As such, the ability to combine height reconstruction with the inherent advantages of the helium probe would provide a unique tool for the purposes of taxonomy.

Stereophotogrammetry is an imaging technique whereby height can be recovered through the collection of multiple micrographs, each at a different perspective (observation angle), with feature heights calculated from the resultant lateral (sometimes referred to as parallax) shifts. Here we present the development and implementation of stereophotogrammetry in the SHeM by means of tilting the sample with respect to the helium beam, accomplished via a compact, multi-axis sample mount. The mount’s performance was first benchmarked against a calibration standard, before the ‘3D SHeM technique’ was applied to a range of taxonomic samples. The resulting quantitative biometrology data highlights the extended capabilities of the SHeM, whilst retaining the inherent advantages of a non-destructive, low energy probe.

## Principles

**Figure 1:**
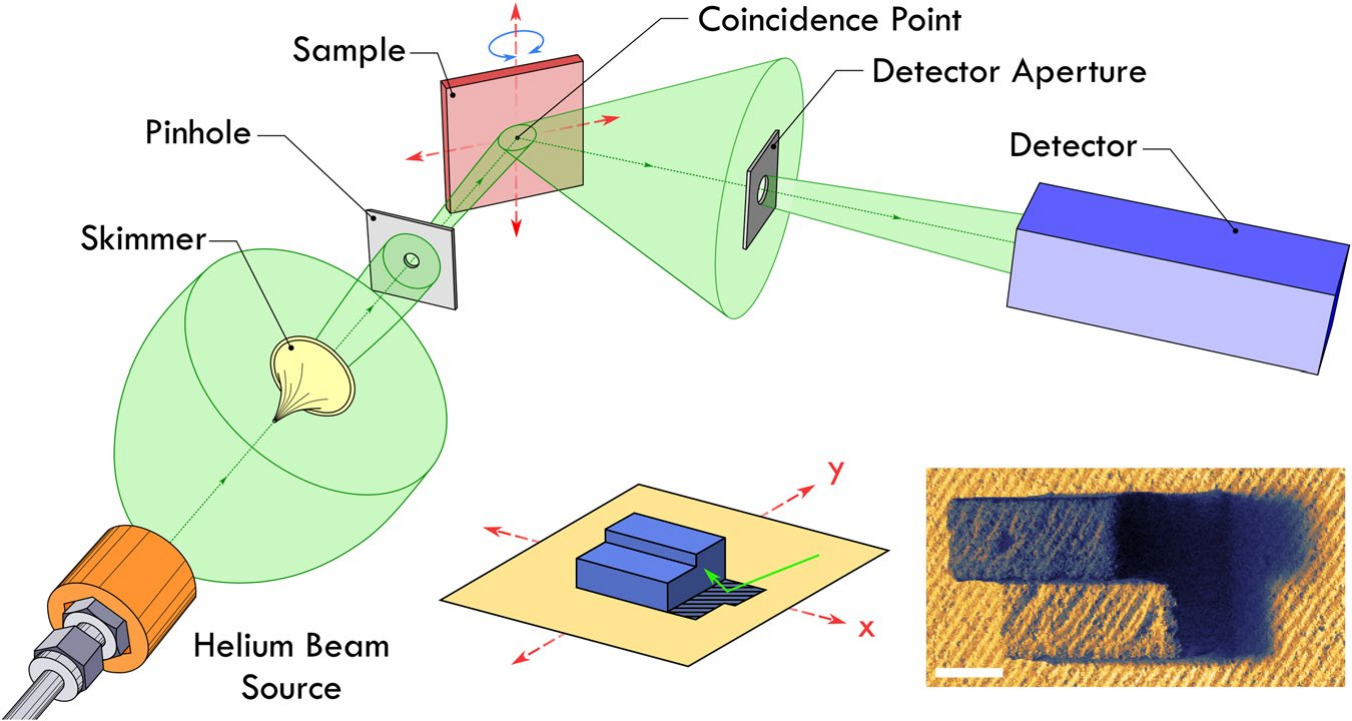
Schematic diagram (not to scale) illustrating the path of helium through the SHeM. The neutral helium atom beam generated by the free-jet expansion is progressively collimated by the skimmer and pinhole apertures, resulting in a pencil beam that strikes the sample surface. The intensity of backscattered helium atoms passing through the detector aperture (located to collect specular reflections) is recorded as the sample is rastered in two dimensions underneath the beam (see red arrows). Insets show an illustrative example of image formation in the SHeM, highlighting the effects of both occlusion and projection distortion by means of a simple geometric sample. The final produced SHeM micrograph of a 3D-printed analogue of the sample is also shown for completeness. Scale bar 500 *μ*m, 2.6 seconds dwell per pixel.

The schematic shown in Fig. 1 presents the basic layout of the SHeM (described in detail in the literature^22^). The helium atom beam is produced via a free-jet expansion; a process in which a dense volume of gas is forced to expand through a nozzle into high vacuum^26,27^. A narrow portion of the helium beam is selected out by the use of progressively smaller apertures, namely the skimmer orifice and final pinhole, which defines the lateral resolution of the instrument via the projected spot size onto the sample. The produced pencil beam of atoms then strikes the sample surface, with the backscattered intensity dependent on the local topography and chemical composition^23,24^. A fraction of the backscattered atoms is selected by the detector aperture and the partial pressure of helium within the subsequent volume is sampled via a standard mass spectrometer. By rastering the sample under the beam in two dimensions, and monitoring the detected count rate at each position, an image of the sample surface can be generated. A detailed discussion of the experimental setup is given in the Materials and Methods section.

The SHeM has a 90° scattering geometry; that is, the beam is incident on the sample at 45°, with the detector monitoring the helium that scatters specularly from the surface. The standard imaging position is defined by the coincidence point located at the intersection of the incoming and outgoing beams (see Fig. 1). This particular scattering geometry has specific consequences for image formation in the instrument^24^. Much like in secondary electron SEM images, the image perspective (viewing angle of the sample) is set by the incident beam angle, whilst the micrographs themselves appear to be illuminated from the detector^28^. Occlusion of the detector or the beam will respectively lead to shadowing or masking in the resultant micrograph^24^. Additionally, the relative tilt of the incident beam with respect to the sample leads to projection distortion (namely variations in the apparent length of features along the tilted axis)^24,28^. Insets in Fig. 1 show a render and a SHeM micrograph of a sample with beam and detector positions overlaid. The micrograph demonstrates examples of occlusion and projection distortion.

### Stereophotogrammetry in SHeM

Stereophotogrammetry requires a minimum of two component images taken at different perspectives to measure height. As with other scanned beam microscopies, the perspective in a SHeM micrograph is determined by the incident beam angle. From the component images, the apparent lateral shift of specific features is used to calculate their height difference; thus height resolution from stereophotogrammetry is, in principle, limited only by the lateral resolution of the instrument^29^. A more complete description of the instrument’s resolution can be found in the Materials and Methods section.

The SHeM has a fixed beam-detector geometry (see Fig. 1), and therefore the sample itself must be tilted relative to the beam to obtain the required change in perspective. As a consequence of the fixed geometry, the detector position will change in lockstep with the beam, relative to the sample plane. The varying detector position results in an apparent change in the illumination of the sample, causing a movement of any masking present within the component images. The change in illumination thus reduces the number of features which can be used to determine height. Similarly, the change in perspective can lead to low-lying features being occluded, again reducing the information available for height reconstruction. Nevertheless, limiting the tilt angle between component images, and increasing the number of component images captured, maximises the density and accuracy of the reconstruction.

**Figure 2:**
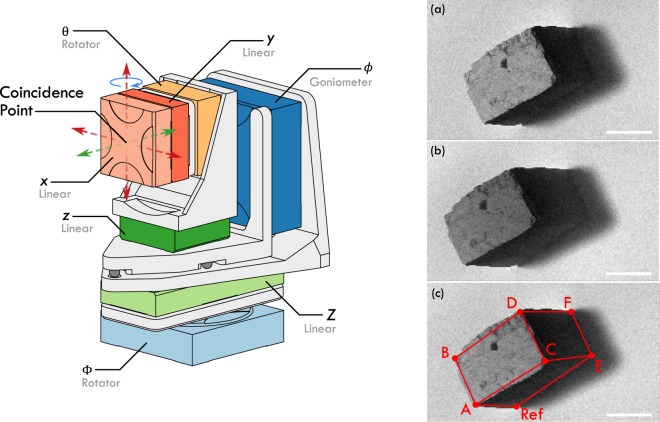
Schematic representation of the stereo-mount developed for the SHeM. Coloured sections refer to actuators responsible for the specific degrees of freedom needed to conduct routine scan operations and the rotations necessary for stereophotogrammetry. The *x* & *y* actuators are used for the rastering action to form the image (red arrows), the *z* actuator is used to position the sample at the coincidence point prior to imaging (green arrows), *ϕ* is the goniometer used to tilt the sample with respect to the beam for stereophotogrammetry (blue arrow); all remaining actuators (*θ*, Φ, & *Z*) are used to align the sample with respect to the optical system (Fig. 1), and to aid sample exchange. Insets (**a**), (**b**), and (**c**) show micrographs of a pyrite crystal captured with varying incident beam angles; (**c**) also includes an overlaid wireframe whose dimensions are derived in Appendix 1: Stereophotogrammetry. Scale bar 1 mm, 1.85 seconds dwell per pixel for all micrographs.

The additional angular degree of freedom required for stereophotogrammetry is provided by a seven-axis sample mount, highlighted in Fig. 2. The stereo-mount employs closed-loop slip-stick actuators mounted to custom brackets. Details of the installed actuators and their corresponding movements are listed in Table 1 in Materials and Methods. Support brackets were designed for both ultra-high vacuum operation and stability (to minimise the effect of vibrations during imaging). The tilting action (*ϕ*) is provided by a goniometer, positioned behind the sample. The goniometer is positioned on the stereo-mount such that the sample and rastering axes (*x* & y actuators) rotate about a vertical axis through the coincidence point (Fig. 2). This goniometer position minimises any undesired translational shifts between component images, thus maximising the available field of view. It should be noted that as the rastering axes rotate with the sample, the subsequent analysis is simplified as compared to other applications of stereophotogrammetry - see Materials and Methods^30,31^.

The stereo-mount was characterised by performing stereophotogrammetry on a highly simplified sample of known geometry (pyrite crystal). Component images were collected at two incident beam angles as displayed in insets (a) and (b) in Fig. 2. A sparse reconstruction consisting of 3D coordinates of key features on the sample surface was derived using the procedure outlined in Materials and Methods (represented as a wireframe diagram in Fig. 2(c)). The calculated coordinates were compared against measurements taken with a confocal laser scanning microscope and good agreement was found between the two techniques (see Materials and Methods). With the accuracy of the reconstruction confirmed, the ability of the 3D SHeM technique to provide quantitative biometrological imaging without risk of damage or morphological alteration was highlighted using two taxonomic case studies: (1) flora (*Arabidopsis thaliana*) and (2) fauna (*Heterodontus portusjacksoni*).

## Results and Discussion

### Case study I - *Arabidopsis thaliana*

Trichomes are hair-like epidermal structures located on the aerial tissues of a wide range of plants, algaes, and lichens^32^. By virtue of their diverse physical properties, trichomes fulfill a number of different roles, including: protection from insect damage; a deterrent to herbivores; protection from heat, frost, and abrasion; moisture conservation; secretory functionality; and even predatory behavior^33–36^. Trichomes have long been considered vital in comparative studies of plant species, with more than 300 structural descriptions in the botanical literature characterising various morphological types^33^. The basic presence of the structures allows differentiation between species (and in some instances, a determination of the age of the plant), whilst the size, location, and shape of the trichomes are important diagnostic characteristics in plant taxonomy^34–36^. Thus, studies of leaf micromorphology can aid in plant identification and benefit phylogenetic studies.

**Figure 3:**
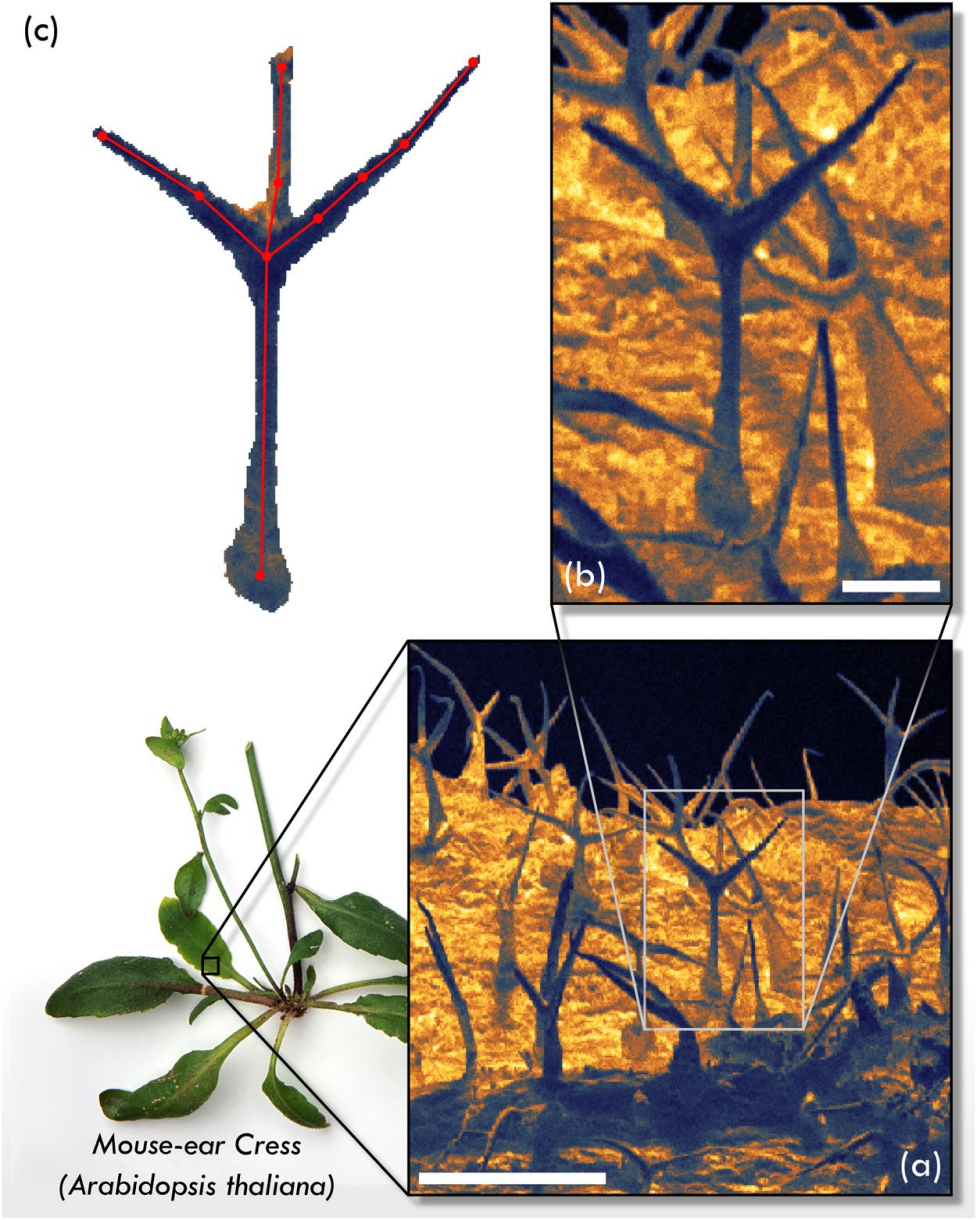
SHeM micrographs of the trichomes appearing on the adaxial surface of a rosette leaf belonging to a sample of *A. thaliana*. (**a**) Wide area region of the sample, displaying many trichomes emerging from the leaf surface. Scale bar 500 *μ*m, 2.6 seconds dwell per pixel. (**b**) Zoomed in region highlighting a single trichome as indicated by the box inset in (**a**). Scale bar 100 *μ*m, 2.6 seconds dwell per pixel. (**c**) Points of interest from the micrograph shown in (**b**) that were monitored when performing the 3D coordinate reconstruction via stereophotogrammetry. This figure is not covered by CC BY licence. [Photograph of plant specimen is credited to Neil Fletcher/Getty Images]. All rights reserved, used with permission.

For the purposes of demonstrating the 3D SHeM technique, the trichomes on the leaves, stems, cauline leaves, and sepals of *A. thaliana* (mouse-ear cress) are ideal candidates, as they are widely acknowledged as one of the most studied model plant species^37–39^. *A. thaliana* allows plant development and differentiation to be studied in terms of the smallest unit of an organism, namely a single cell. As a result of their accessibility, size, rapid growth, and non-essential nature^34,39^, trichomes are an excellent candidate for investigating the effects of editing their genetic architecture. Wild-type plants develop single cell, non-glandular trichomes across much of the plant epidermis, with those on the rosette leaves developing into the classical three-branched (dendritic) pattern associated with the species. The adaxial surface of a rosette leaf from a mature *A. thaliana* sample (previously dried and stored for more than 50 years) were imaged in the SHeM, with the results shown below in Fig. 3.

Figure 3(a) shows a wide area (some 1500 *μ*m square) SHeM micrograph of the *A. thaliana* leaf, including a large number of trichomes protruding from the epidermis. One notable feature of micrographs generated by the instrument is a large field of view; that is, the ability to move from wide area scans all the way down to fine detail whilst retaining a sharp image. For example, Fig. 3(b) shows a zoomed region of the sample appearing in Fig. 3(a), highlighting an individual trichome with the typical three branched morphology. These instrument properties allow us to see not only the features of individual trichomes, but also the patterning of the structures across the surface of the leaf. Note that the information in the micrographs is produced exclusively from the surface of the sample due to the nature of the helium atom-surface interaction. Additionally, there exists no potential for sample charging, even without any form of sample preparation.

The trichome in Fig. 3(b) was the subject of a stereophotogrammetric study, resulting in the generation of a sparse 3D map of the structure. The features of interest for the purposes of the reconstruction - namely, easily identifiable points on the body of the trichome - are overlaid on the micrograph in Fig. 3(c).

**Figure 4:**
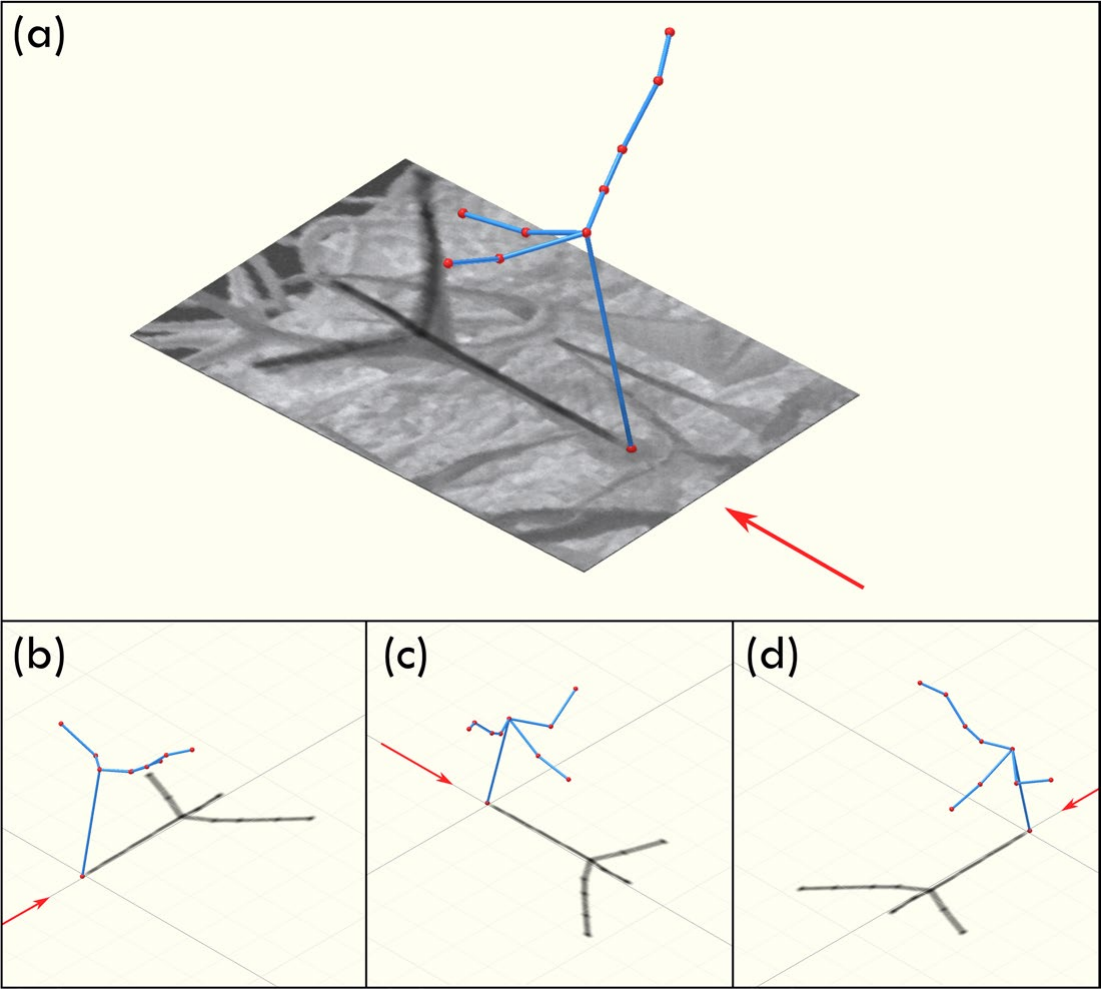
3D wireframe model of a trichome as extracted via stereophotogrammetry, overlaid over the SHeM micrograph from which the coordinates were extracted. Note that, to guide the eye, the model has been lit in the same manner as the incident beam in the SHeM, thus resulting in the produced shadow (ie. the mapping of the 3D object down to a 2D plane) closely matching the observed features. Arrow indicates the direction of the incident helium beam. Insets (**b**), (**c**), and (**d**) show the wireframe model from alternate angles to highlight the 3D topography of the trichome branches.

In order to better interpret the 3D coordinates derived from the reconstruction, a wireframe representation of the trichome was generated (Fig. 4). The prototypical arrangement of the trichome for a wild-type specimen comprises an equidistant spacing of each of the branches^34^. On first inspection, the trichome in the SHeM micrograph shown in Fig. 3(b) would seem to fit the standard description; however, the 3D SHeM technique reveals otherwise. As highlighted by the different rotational views in Fig. 4, all three branches of the trichome can be seen to lay on one side of the main stem, with the appearance in the micrograph a result of the image formation process in the SHeM^24^. The arrangement of the branches is likely a consequence of the method used for preservation of the sample, namely being dried and pressed between sheets of tissue for long term storage.

A major aim of developmental biology is to understand how the three-dimensional morphology of organisms arises through base level molecular and cellular mechanisms^40^. Increasingly, the 3D properties of a sample that may be inferred from standard 2D imaging are not sufficient. As highlighted by the reconstruction of the *A. thaliana* trichome, quantitative 3D data are required to further the field^40^. More broadly, the distribution of the trichomes across the surface of a leaf is critical, with the density and patterning confirmed to be a variable of selective importance to natural populations^40,41^. Research groups have been attempting to generate 3D surface reconstructions of young, curved leaves, in order to determine the distances between trichome locations to gain insight into the genetic markers that regulate their patterning^34,41,42^. Just as the specific size and shape of a single trichome could be extracted as in Figs 3 and 4, the work here shows that stereophotogrammetry in the SHeM can be extended to generate 3D maps of trichome distributions.

### Case study II - *Heterodontus portusjacksoni*

A unique aspect of the taxonomic sub-classes *Elasmobranchii* (sharks, skates, and rays) and *Holocephali* is that they exhibit a complete or partial coverage of their skin with dermal denticles (placoid scales or ‘skin teeth’). These tiny scales covering the animal are unique, tooth-like structures embedded into the deeper collagenous layer of the skin. Dermal denticles can have various functions including (amongst others): protection from predators, ectoparasites (skin parasites), and epibionts (non-parasitic organisms which live on the surface of the host); a reduction of mechanical abrasion; or a lowering of swimming-induced drag^43–45^. In addition to a wide range of overall sizes and aspect ratios, the denticles can exhibit various crown shapes linked to their biological function. Indeed, the denticle shape can vary across the body of an individual animal, as well as with species^44,45^.

Historically, morphological investigation of the dermal denticles of sharks have been used to taxonomically classify different families and to yield ecological information about shark communities^45–47^. These epidermal features have recently seen increased interest due to access to high resolution imaging techniques, allowing for quantitative measurements and a more complete approach to the characterisation of the animals. Beyond reasons of classification, the structure (and thus function) of shark skin is also of interest to biomimetics, the study of naturally occurring properties of plants and animals for the purpose of inspired design^48,49^. In the specific case of shark skin, strong interest has been shown in replicating the denticle riblets aligned to the direction of fluid flow to produce drag-reducing competition swimsuits and plastic coatings for aircraft^48,50^.

**Figure 5:**
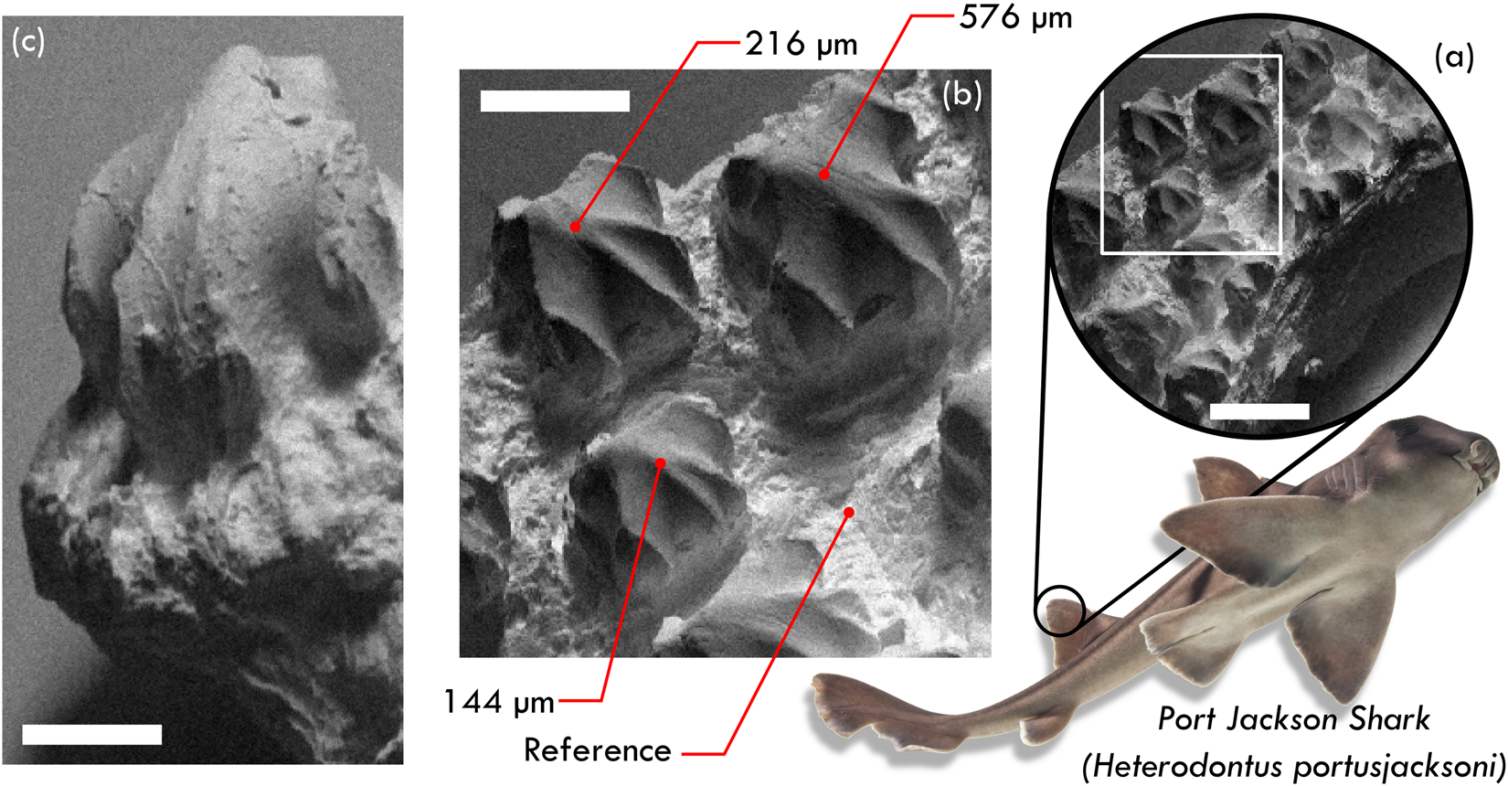
SHeM micrographs of the dorsal skin obtained from a female Port Jackson shark. (**a**) A ca. 3 × 4 mm section of skin, including several dermal denticles protruding out of the underlying surface. Scale bar 1 mm, 1.7 seconds dwell per pixel. (**b**) Zoomed region of the same area of tissue, indicated by the box inset from (**a**). Micrograph focuses on three dermal denticles, which exhibit the characteristics of defensive functionality^45^. Annotations refer to the heights of specific features as obtained through the 3D SHeM technique; heights of (144 ± 72) *μ*m, (216 ± 72) *μ*m and (576 ± 72) *μ*m were found for the various denticles, as referenced to a plane across the general skin surface. Scale bar 500 *μ*m, 1.7 seconds dwell per pixel. (**c**) Micrograph of an additional lone denticle from a different region of dorsal skin, serving to highlight the intuitive nature of the images produced by the technique. Scale bar 250 *μ*m, 1.7 seconds dwell per pixel. This figure is not covered by CC BY licence. [Photograph of Port Jackson shark is credited to Frank Greenaway/Getty Images]. All rights reserved, used with permission.

The dermal denticles on the dorsal fin of a female Port Jackson shark (*H. portusjacksoni*) were imaged in the SHeM, and are presented in Fig. 5. In particular, Fig. 5(a) shows a ca. 3 × 4 mm section of the shark skin exhibiting an array of dermal denticles. Despite the high aspect ratio of the imaged denticles, the details in the micrographs (Fig. 5(b,c)) remain sharp over millimetres in depth, width, and length; highlighting the instrument’s large depth of field (see Materials and Methods) in combination with its wide field of view. The instances of occlusion in the micrograph - for example, towards the base of the lone denticle shown in Fig. 5(c) - adds to the intuitive nature of the produced micrographs and emphasises the complex texture of both the denticles and the underlying skin.

The specific crown shape of the observed denticles are a good initial indication of their basic functionality. The denticles observed in the SHeM micrographs in Fig. 5 have a number of characteristic features which allow them to be categorised according to the work of Dillon *et al*.^45^. The denticles can be described as having a diamond, square or arrow shape, and their size (namely width and length) is of the order of several hundred micrometers; rather large in the context of denticle classification. The micrographs show several ‘subparallel’ complete ridges on each denticle, leading towards a single, round-shaped peak. From Fig. 5(c) it can be deduced that there are no microstructures on the surface of the denticles. All of the aforementioned features are characteristic for either ridged abrasion strength (allowing the shark to resist damage while searching the seafloor for food) or defensive functionality (specifically, warding off the settlement of ectoparasites and epibionts). The significant overlap between these two categories has been described previously^45^; distinguishing between the categories requires quantitative assessment of the denticle features.

The 3D SHeM technique provides us with taxonomical data extending beyond widths and lengths; in particular, the specific heights and aspect ratios of the denticles can now be quantified. In the case of the dorsal fin denticles shown in Fig. 5, various denticle heights (up to a maximum of 580 μm) were found above a reference plane representative of the underlying skin surface. The extracted values allowed us to determine the crown thickness ratios (defined in Dillon *et al*.^45^ as √(*crown length* × *crown width*)/*crown thickness*), which were evaluated to be between 1.2 and 3.8 - well matched to the literature values for both ridged abrasion strength and defensive functionality^45,51^. The critical point of differentiation for the defence category is that the denticle crowns possess one highest peak, with the main cusp pointing in an upward-posterior direction. From the dimensions extracted from the micrographs in Fig. 5, the presence of a local maxima in the heights of the crown peaks for each denticle was confirmed. The quantitative data obtained via the 3D SHeM technique thus provides the final, conclusive evidence that the imaged structures belong to the functional group of defensive denticles^45,51^.

It is known from the literature that although the skin of demersal shark species, like the *H. portusjacksoni*, is mostly covered in denticles for reasons of abrasion strength^51^, defensive functionality denticles have also been observed for those inhabiting muddy or sandy substrates^45,51^. Through the use of stereophotogrammetry, the subtle differences between denticle categories can be exploited for a more complete classification. Such categorisation shows that not just qualitative, but comprehensive quantitative assessments of taxonomic features is vital; a focus of recent literature concerning *Elasmobranchii*^45,51^. Indeed, the case study presented here demonstrates that the 3D SHeM technique offers a promising addition to the repertoire of tools available for the classification of species.

### Future outlook

At present, the very highest quality micrographs require relatively long imaging times; for example, the images presented in both Figs 3 and 5 involved collection times of up to 2.5 days. However, the low energy of the helium probe, combined with the stability of the measurement system, ensures that the integrity of the sample is preserved over the length of the experiment. Specifically, long dwell times under the neutral helium beam do not induce any form of radiation damage or sample heating, thus mitigating one of the drawbacks of extended measurements. Whilst the helium probe itself presents no risk of sample damage, the requirement for a high vacuum environment presently limits the SHeM from imaging samples such as live cells or hydrated specimens.

Work is currently underway across the field of neutral atom microscopy to address these issues. Reducing the imaging time for the instrument requires an increase in the available helium signal, thus motivating interest in higher sensitivity detectors^52,53^, neutral atom optics^17,54^ and optimization of source designs^55^. Increased signal levels also offer the potential for improved spatial resolution, as well as the addition of sufficient differential pumping to facilitate the use of an environmental sample chamber. An alternative approach to the latter would be to take advantage of recent work by Wojcik *et al*.^56^ involving the application of a conformal graphene sheet to preclude desiccation under the high vacuum environment. Either approach opens the technique up to an even broader array of samples. Such modifications would further enhance the applicability of neutral helium microscopy to biological metrology, beyond the inherent advantages of a non-damaging probe with minimal sample preparation requirements.

## Conclusion

Neutral helium microscopy is an emerging imaging technique, attractive for a wide range of delicate samples that could otherwise be damaged under the energetic probes of established microscopies. Biologists working to describe the myriad of species are increasingly required to employ techniques able to produce quantitative data from their samples in order to accurately and unambiguously classify them. We have described the principles of stereophotogrammetry as applied to the SHeM, a process which enables height data to be extracted from the collected micrographs. Furthermore, the derivation of feature heights ultimately allows for the generation of sparse 3-dimensional maps of the sample surface. We have highlighted the potential of the technique through case studies of both flora (*A. thaliana*) and fauna (*H. portusjacksoni*), and thus demonstrated the capabilities of the 3D SHeM technique as an exciting complementary tool for the purposes of taxonomy.

## Materials and Methods

### Sample Preparation - Geometric Shape

A sample consisting of a simple geometric shape (see Fig. 1) was designed using Autodesk Inventor 2016 and printed using a Formlabs Form 2 stereolithography 3D printer (RS-F2-GPCL-04 resin, 2.5 μm steps). The 3D printed structure was washed in analytic grade (99.8%) isopropyl alcohol and blow-dried with nitrogen before affixed to a sample slide using an adhesive carbon tab.

### Sample Preparation - Calibration Slide

A sample of pyrite, sourced from a private collection, was affixed to a sample slide using an adhesive carbon tab.

### Sample Preparation -* Heterodontus portusjacksoni*

A sample of the dorsal fin was taken post mortem from a freshly deceased female *H. portusjacksoni* shark at the Sea Life Sydney Aquarium. The fresh dorsal fin sample was placed in formalin for transport and storage. Skin samples were prepared for SHeM imaging using the following procedure: two ca. 4 × 3 mm sections of the dorsal fin’s skin were dissected with a scalpel, rinsed and washed several times in deionised water, followed by blow-drying with nitrogen gas. The samples were then stuck down via an adhesive carbon tab, before being entered into a vacuum chamber of ca. 5 × 10^−2^ mbar for ca. 12 hours to ensure the removal of residual formalin.

### Sample Preparation - *Arabidopsis thaliana*

*A. thaliana* samples were obtained from the reference collection belonging to the Royal Botanic Gardens and Domain Trust, National Herbarium of New South Wales (Collection Numbers NSW707669 and NSW939449). A single leaf from NSW707669 was stuck to a SHeM sample slide via an adhesive carbon tab, before being entered into the microscope sample chamber.

### SHeM Imaging Conditions

All SHeM micrographs collected for this study were generated under the following conditions. The helium source (fitted with a 10 μm nozzle) was operated at 200 bar and 297 K, with the resultant free-jet expansion progressively apertured by a skimmer (100 μm diameter, located 17 mm downstream of the source’s nozzle) and a pinhole (5 μm diameter, positioned a further 68 mm downstream of the skimmer). The working distance (distance from the pinhole to the sample) was set to 2.9 mm, with the backscattered atoms collected by a detector aperture (1 mm diameter) also located 2.9 mm from the sample. The skimmer and pinhole arrangement chosen for the present studies yields a Gaussian beam with a full width at half maximum of (6.9 ± 0.2) μm at the working distance as above. This instrument setup leads to a ca. 3 μm lateral resolution based on the Dawes’ criterion^57^. The minimum resolvable step height observable in a single SHeM image is (67 ± 0.5) μm^24^ and the depth of field is 4.3 mm, as calculated using the Rayleigh length of the instrument. During imaging, the sample chamber pressure was typically of the order 1 × 10^−8^ mbar. All micrographs were collected with the sample goniometer set to 0°, unless otherwise specified.

### 3-Dimensional Reconstruction

The imaging process in the SHeM involves a transformation from object (3D) to image (2D) space. This mapping creates an ambiguity over movement in either the *x* or *z* axes, or some combination of the two (see Appendix 1: Stereophotogrammetry). By taking a set of micrographs at multiple tilt angles and comparing the apparent lateral shift between the component images in the set, the relative heights of specific features can be recovered. In practise, this method first requires the images to be ‘rectified’ - a process by which any translational offsets between the images in the set are accounted for by aligning them all to a common point of reference. Subsequently, the pixel shift(s) for a fixed feature within two or more micrographs are compared; these shift(s) yield the relative heights according to the theory presented in Appendix 1. With the heights of the feature recovered via the stereophotogrammetry method, we now possess sufficient information to generate the 3D coordinates of the feature, relative to the common point of reference. This process is further elaborated upon in Appendix 1.

### Stereo-mount Calibration

The stereo-mount was calibrated by reconstructing the geometry of a pyrite crystal sample. A set of three component images were taken, with incident beam angles of 39°, 45° and 50°, and rastering steps of 20 μm. The 39° and 50° images were used to measure the relative height of a number of features on the crystal, whilst the 45° image was used to derive the 3D coordinates of said features. Comparative coordinate measurements were taken using an Olympus FV1000 confocal laser scanning microscope in reflection mode (4 µm z-slices, 635 nm laser)^58^. Measurements taken using either technique were found to be in good agreement within experimental uncertainty. See Appendix 1 for the full derivation of the 3D coordinates of the pyrite crystal using the SHeM images and the comparison to the confocal measurements.

### Stereo-mount Axes and Actuators

Table 1 lists details of the installed Stereo-mount actautors and their corresponding movements.

**Table 1:** Seven-axis stereo-mount description. All actuators are ‘attocube industrial line’ and were purchased from attocube systems Inc.

Name	Movement	Part Number
x	linear (raster x)	ECS3030/UHV/NUM+
y	linear (raster y)	ECS3030/UHV/NUM+
z	linear	ECS3030/UHV/NUM+
*ϕ*	goniometer	ECGt5050/UHV/NUM
Φ	rotator	ECR5050/UHV/NUM
θ	rotator	ECR3030/UHV/NUM+
Z	linear	ECS3070/UHV/NUM

## Electronic supplementary material

Appendix 1

## Data Availability

The datasets generated during and/or analysed during the current study are available from the corresponding authors on reasonable request.
