# Supplementary material for: Taxonomy through the lens of neutral helium microscopy
Source: Sci Rep. 2019 Feb 14;9:2148. doi: 10.1038/s41598-018-36373-5 (PMC6375913; doi:10.1038/s41598-018-36373-5)
Supplement: Supplementary file 1 — Appendix 1 [file 41598_2018_36373_MOESM1_ESM.pdf]

Supplementary Information

Taxonomy through the lens of neutral helium microscopy

Thomas A. Myles[1,†], Sabrina D. Eder[1,2,†,*] , Matthew G. Barr[1], Adam Fahy[1], Joel Martens[1], & Paul C. Dastoor[1,*]

[1] Centre for Organic Electronics, University of Newcastle, Callaghan, New South Wales 2308, Australia
[2] Department of Physics and Technology, University of Bergen, Allégaten 55, 5007 Bergen, Norway
[*] corresponding: sabrina.eder@uib.no, paul.dastoor@newcastle.edu.au
[†] these authors contributed equally to this work

Supplementary Information

Appendix 1: Stereophotogrammetry

Image Formation:

Similar to photography[1] and scanning electron microscopy (SEM)[2], the imaging process in a scanning helium microscope (SHeM) involves mapping points in 3D space (object space) onto a 2D image (image space). Like in SEM, the transformation from object space to image space is governed by the incident beam (see Fig. A)[3], where points in object space (*a*, *b*, *c*, & *d*) are projected along the direction of the incident beam onto the image plane (*a'*, *b'*, *c'*, & *d'*), which is parallel to the rastering axes (*x* & *y* actuators). The exact definition of the coordinate axes in both image (***u***, ***v***) and object space (***x***, ***y***, ***z***) is arbitrary, and as such the transformation from object to image space is not unique. However, there are a natural set of axes that arise from the rastering actuators of the stereo-mount and the incident beam, which simplify this transformation. If we align the image axes (***u***, ***v***) with the *x* and *y* actuator axes, as seen in Fig. 1 & 2, the ***u*** axis will point left-to-right and the ***v*** axis points bottom-to-top. As such, the rastering motion used to form an image involves independently stepping along the ***u*** & ***v*** axes, respectively. Additionally, if we define the ***x*** & ***y*** axes in object space to be similarly aligned with the *x* & *y* actuators, then a movement along either of these axes produces an equivalent motion in the image's ***u*** & ***v*** axes. We define the final ***z*** axis to be perpendicular to the *x* & *y* actuators, pointing 'outwards' as shown in Fig. A. With the object axes defined as such, the incident beam is entirely within the ***xz***-plane, tracing a line along the $[-\cos\theta_i, 0, -\sin\theta_i]^T$ direction, where θ_i is the angle measured between the ***z*** axis and the incident beam (see Fig. A).

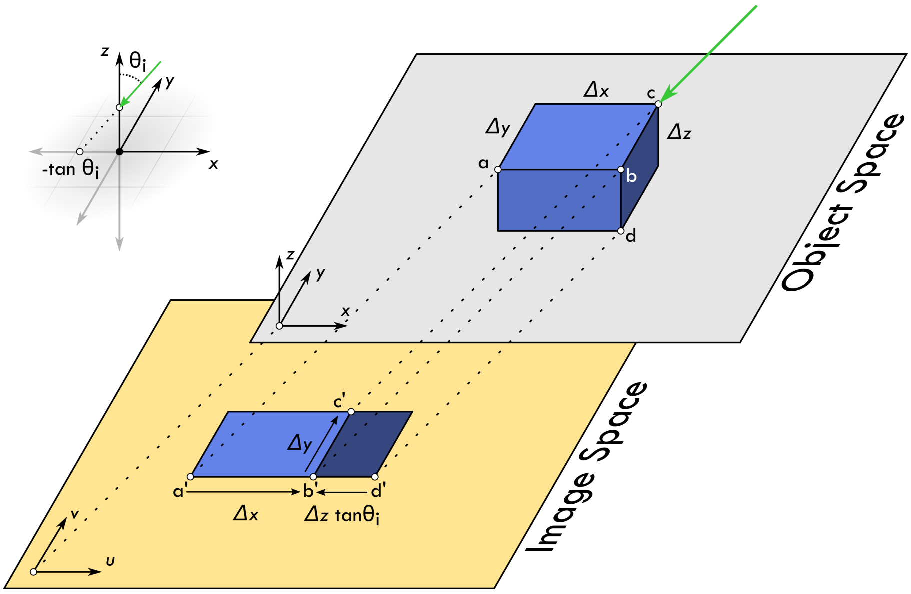

Figure A: The transformation of a point from object space (grey) into image space (tan) is dependent upon the incident helium beam (green arrow). For the geometry of the SHeM, a translation in the ***x*** axis ($a \to b$) or ***z*** axis ($d \to b$) produces a corresponding horizontal (***u***) shift in the image ($a' \to b'$ and $d' \to b'$, respectively). Conversely, a translation in the **y** axis ($b \to c$) produces a corresponding vertical (***v***) shift in the image ($b' \to c'$). Top left inset: points which are collinear with the incident beam will appear at the same position in the image.

With the object and image axes now defined, we need to derive the transformation from the object space to the image space. Consider an infinitesimal translation of a point along the ***x*** axis $a \to b$ in Fig. A; as the ***x*** and ***u*** axes are aligned there is a one-to-one translation in the ***u*** axis and no change in the ***v*** axis ($a' \to b'$). Similarly, for an infinitesimal translation along the ***y*** axis $b \to c$, there is also a one-to-one translation in the ***v*** axis, and no motion in the ***u*** axis ($b' \to c'$). To derive the effect of a translation in the ***z*** axis, we use the incident beam to find the equivalent translation in the ***x*** & ***y*** axes, which we can then relate to a translation in the image plane. Consider an infinitesimal translation along the ***z*** axis from the origin $[0, 0, \delta z]^T$, we now project this point along the direction of the incident beam onto the ***xy***-plane (see Fig. A inset) to find an equivalent point at $[-\tan\theta_i, 0, 0]^T$. As such, an infinitesimal translation in the ***z*** axis $d \to b$ results in a translation scaled by a factor of $-\tan\theta_i$ in the image's ***u*** axis ($d' \to b'$). We can express this mapping in matrix form as follows:

Supplementary Information

$$\begin{bmatrix} u \\ v \end{bmatrix} = \begin{bmatrix} 1 & 0 & -\tan\theta_i \\ 0 & 1 & 0 \end{bmatrix} \begin{bmatrix} x \\ y \\ z \end{bmatrix}. \quad \text{(Eq 1)}$$

For clarity, we maintain linearity and define the origin of the image space to be the projection of the object space's origin, i.e. $[0,0,0]^T \rightarrow [0,0]^T$. The location of the object space origin is arbitrary, and can hence be defined specifically for each experiment.

The transformation from object (3D) space to image (2D) space necessarily involves some loss of information, as the number of dimensions is reduced from three to two. In the case of the SHeM, this loss presents itself as an ambiguity over whether a translation in the image's ***u*** axis constitutes a movement in either the ***x*** or ***z*** axes, or some combination of the two. This ambiguity can be removed if the translation in either the ***x*** or ***z*** axes is known. Stereophotogrammetry allows us to directly measure the ***z*** translation of a point of interest, and thus obtain a unique solution for its position in object space.

Height Reconstruction (Stereophotogrammetry):

As the projection of features onto the image plane (Eq. 1) changes with the incident beam angle (θ_i), it is possible to recover the relative height (***z*** translation) information lost in the imaging process by taking multiple images with varying θ_i (component images). Consider some arbitrary feature located in object space $[x_f, y_f, z_f]^T$, which has been imaged twice, once with an incident beam angle of θ_{i1} and then again with an incident beam angle of θ_{i2}. This feature will appear at different positions within each image; being located at $[u_1, v_1]^T$ and $[u_2, v_2]^T$ within the first and second component images, respectively. Note that due to the SHeM beam geometry, the ***v*** coordinate of the features in question will not change between the two images (resulting in $v_1 = v_2$).

If we write the expressions for the ***u*** coordinate of the feature in each image, and subtract the two, we can derive an expression for z_f:

$$u_1 = x_f - z_f \tan\theta_{i1},$$

$$u_2 = x_f - z_f \tan\theta_{i2},$$

$$h = z_f = \frac{u_1 - u_2}{\tan\theta_{i2} - \tan\theta_{i1}}. \quad \text{(Eq 2)}$$

Here we choose to refer to the derived z_f as a relative height (h), as the position of the object space's origin (and hence where z_f is measured from) is arbitrary, and set through a process hereafter referred to as 'rectification'. Rectification involves defining a common point of reference for the set of component images – often a strongly contrasting feature of interest, matched easily across the set. The common point becomes the origin of the object space $[0,0,0]^T$ and therefore will provide the point where image coordinates $[u_1, v_1]^T$ and $[u_2, v_2]^T$ are referenced to. In practice, small misalignments between the sample and the incident beam path will result in the component images appearing to translate relative to each other, as a function of θ_i. For example, potential offsets between the tilting axis, incident beam, and the object space's ***xy***-plane (see Fig. B) will lead to systematic errors in the height reconstruction, if not corrected for. By design of the stereo-mount, these undesired translations will be limited to the ***u*** axis only, and thus can be corrected by a simple linear shift of component images such that the common point of reference is aligned. After rectification has been performed, the relative heights of any features of interest can be derived using Eq. 2.

Supplementary Information

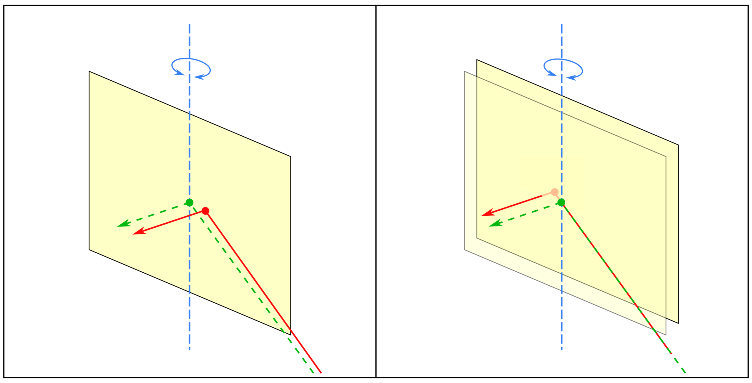

Figure B: Potential misalignments of the ***xy***-plane that would require correction in the process of image rectification. Intended path of the incident helium beam is shown in green, while the resulting beam path subject to misalignment is shown in red. Left: sample axis of rotation not aligned with the position where the beam strikes the sample surface. Right: sample scan plane not positioned at the specular position.

Coordinate Derivation:

With relative height of a feature measured via stereophotogrammetry, the ambiguity between a translation along the ***x*** and/or ***z*** axes is removed, thus enabling the recovery of its 3D coordinates with respect to the common point of reference. Consider a feature displaced from the reference point by a known relative height (h_f), and unknown ***x*** & ***y*** displacements $[x_f, y_f, h_f]^T$. With an incident beam angle of θ_i the feature in question will be located at $[u_f, v_f]^T$ in the image, relative to the common point of reference. To derive the 3D coordinates of the feature in object space, we substitute these values into Eq. 1, and then solve for x_f and y_f:

$$\begin{bmatrix} x_f \\ y_f \end{bmatrix} = \begin{bmatrix} u_f \\ v_f \end{bmatrix} + h_f \begin{bmatrix} \tan\theta_i \\ 0 \end{bmatrix}. \qquad \text{(Eq 3)}$$

By measuring the image coordinates $[u_f, v_f]^T$ within an image taken at $\theta_i = 45°$, the above equation is further simplified. Including the ***z*** coordinate for completeness ($z_f = h_f$ from Eq. 2), we arrive at the following expression:

$$\begin{bmatrix} x_f \\ y_f \\ z_f \end{bmatrix} = \begin{bmatrix} u_f + h_f \\ v_f \\ h_f \end{bmatrix}. \qquad \text{(Eq 4)}$$

With all parameters in Eq. 3 known, we can thus derive the 3D coordinates of any feature relative to the common point of reference.

Worked Example:

To confirm the discussed methodology, a 3D reconstruction of a simple geometric structure (namely a pyrite crystal) was performed. Component images were taken with incident beam angles of 38.3°, 44.3°, and 49.3°, and rastering steps of 20 μm. The θ_i = 38.3° and θ_i = 49.3° images were used to determine relative heights, while the θ_i = 44.3° image was used to derive 3D coordinates. Note that there is a systematic uncertainty of ± 0.2° for all θ_i.

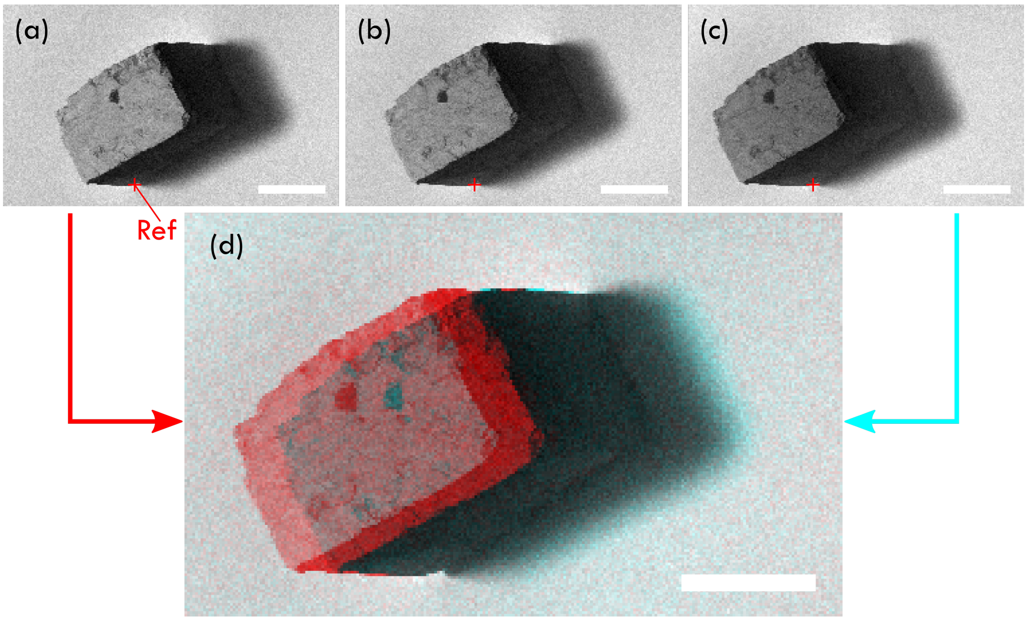

Figure C: The component micrographs of the pyrite crystal collected with an incident beam angle of (a) 38.3°, (b) 44.3°, and (c) 49.3°, and an anaglyph (d) formed from the θ_i = 38.3° (red) and θ_i = 49.3° (cyan) images. Note that one must be careful not to utilise image masks in the rectification of the component set. By way of an example, the cyan tinge across the edge of the masked region does not indicate there is a height difference between the reference feature and the substrate. Rather, this tinge is due to the changing position of the detector aperture relative to the sample, a result of the fixed beam-detector geometry of the SHeM. Scale bar 1 mm, 1.85 seconds dwell per pixel for all micrographs.

First, the reference feature was defined to be lower left corner of the pyrite sample ('Ref' in Fig. C) as this point has strong contrast with the carbon substrate below. The image set was rectified by overlaying the component images with transparencies applied, and the reference feature aligned. As an aside, this overlaid image is typically referred to as an anaglyph when the component images are coloured chromatically opposite as seen in Fig. C. Subsequently, the lateral shift ($u_1 - u_2$ in Eq. 2, and henceforth referred to as the disparity) for each feature of interest (A to F in Fig. D) was measured directly by translating one of the component images (θ_i = 49.3°) until said feature was aligned. This shift away from the original position of the component image is equal to the disparity of that feature. For example, it was found that the θ_i = 49.3° image needed to be shifted 16 pixels right to have feature A appear aligned in the overlaid image; as such feature A possesses a disparity of 16 × 20 μm = 320 μm. Substituting this value for disparity, and the incident beam angles into Eq. 2, the relative height of feature A can be calculated:

$$h_A = \frac{320}{\tan 49.3° - \tan 38.3°} = 858\ \mu m.$$

This process was repeated for all points of interest until a sparse map of heights was formed (Fig. D). The resolution of these height measurements Δh is determined by the smallest resolvable disparity (1 pixel = 20 μm for Fig. C), and the change in incident beam angle (namely, 38.3° to 49.3°). Δh can be calculated using Eq. 2 thusly:

$$\Delta h = \frac{20}{\tan(49.3 \pm 0.2)° - \tan(38.3 \pm 0.2)°} = (53.6 \pm 0.3)\ \mu\text{m}.$$

Supplementary Information

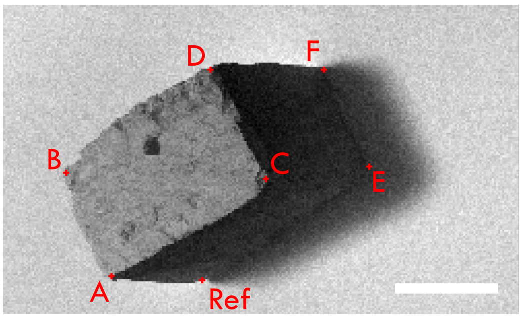

Feature	h (μm)
A	838
B	838
C	891
D	891
E	0
F	0

Figure D: The features of the pyrite crystal, and their respective heights as extracted using stereophotogrammetry. Scale bar 1 mm, 1.85 seconds dwell per pixel. By propagation of uncertainties, all measured heights have an uncertainty of 60 μm.

To extend these height measurements and therefore derive the 3D coordinates of features of interest, the image coordinates of each feature within the θ_i = 44.3° image was measured (see Fig. E.). The uncertainty of these measurements was again taken to be 20 μm (1 pixel).

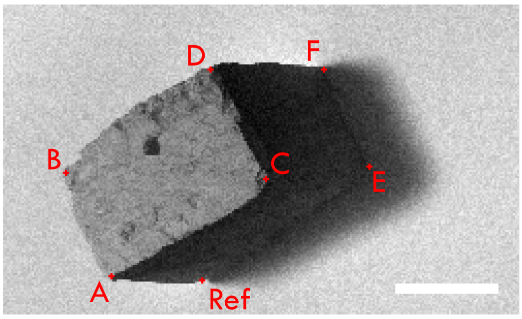

Feature	u (μm)	v (μm)
A	-880	40
B	-1300	1040
C	620	980
D	80	2040
E	1620	1100
F	1180	2040

Figure E: The θ_i = 44.3° image of the pyrite crystal with features marked and their image coordinates. We assume an uncertainty of ±1 pixel for the position of each feature, giving an uncertainty of ±20 μm in each image coordinate measurement. Scale bar 1 mm, 1.85 seconds dwell per pixel.

With both heights and image coordinates measured, the full 3D coordinates of each identified feature can be calculated (Eq. 3). Using feature A as an example:

$$\begin{bmatrix} x_A \\ y_a \\ z_a \end{bmatrix} = \begin{bmatrix} -880 \\ 40 \\ 0 \end{bmatrix} + 858 \begin{bmatrix} \tan 44.3° \\ 0 \\ 1 \end{bmatrix} = \begin{bmatrix} -42 \\ 40 \\ 838 \end{bmatrix}.$$

The evaluated coordinates of each feature were used to generate a wireframe model of the pyrite crystal (Fig. F).

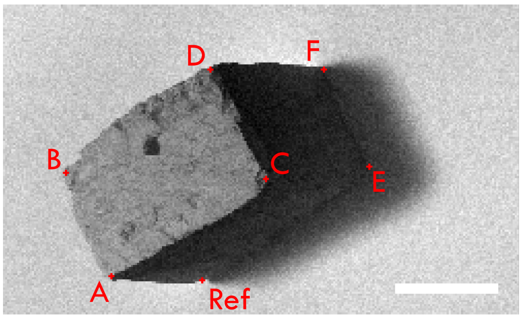

Feature	x (µm)	y (µm)	z (µm)
A	-42	40	858
B	-462	1040	858
C	1510	980	912
D	970	2040	912
E	1620	1100	0
F	1180	2040	0

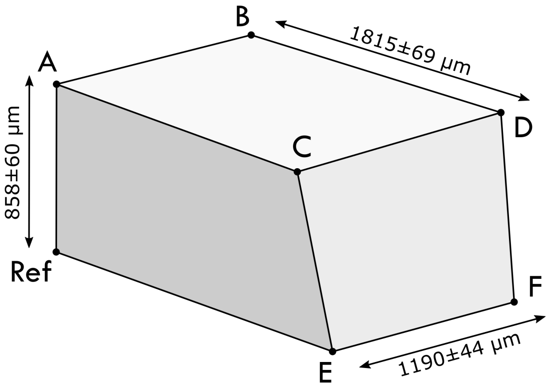

Figure F: The relative 3D coordinates and generated a wireframe model of the pyrite crystal sample. The uncertainty in the ***x***, ***y***, and ***z*** coordinates are ± 56 µm, ± 20 µm, and ± 60 µm respectively. Scale bar 1 mm, 1.85 seconds dwell per pixel.

Comparison to Confocal Laser Scanning Microscopy:

The coordinate measurements of the pyrite crystal derived with the stereophotogrammetry technique were directly compared to those collected using an Olympus FV1000 confocal laser scanning microscope in reflection mode (4 µm z-slices, 635 nm laser). Characteristic dimensions of the pyrite crystal are compared in Table A, demonstrating good agreement between the techniques. Note that all SHeM height measurements are systematically smaller than those found via confocal microscopy. The carbon dot used to adhere the pyrite crystal to the sample slide is covered by an optically-transparent adhesive; the bottom surface of the pyrite crystal is embedded into said adhesive layer. The laser of the confocal microscope can penetrate through this transparent layer, and hence the bottom surface of the pyrite crystal can be seen in the resultant confocal micrographs. However, the adhesive layer is opaque to the surface sensitive helium beam, and thus the bottom surface of the crystal is not visible in SHeM micrographs. Therefore, the lowest points that can be imaged in the SHeM are those defined by the top surface of the adhesive layer, leading to the systematic differences in measured heights.

Supplementary Information

Table A: Comparative distance and height measurements of features taken using both the 3D SHeM technique and an Olympus FV1000 confocal laser scanning microscope.

Measurement	3D SHeM Technique (µm)	Confocal (µm)
AC	1815 ± 69	1880
CD	1190 ± 44	1262
AD	2242 ± 44	2316
h_A	858 ± 60	928 ± 4
h_B	858 ± 60	936 ± 4
h_C	912 ± 60	992 ± 4
h_D	912 ± 60	1016 ± 4

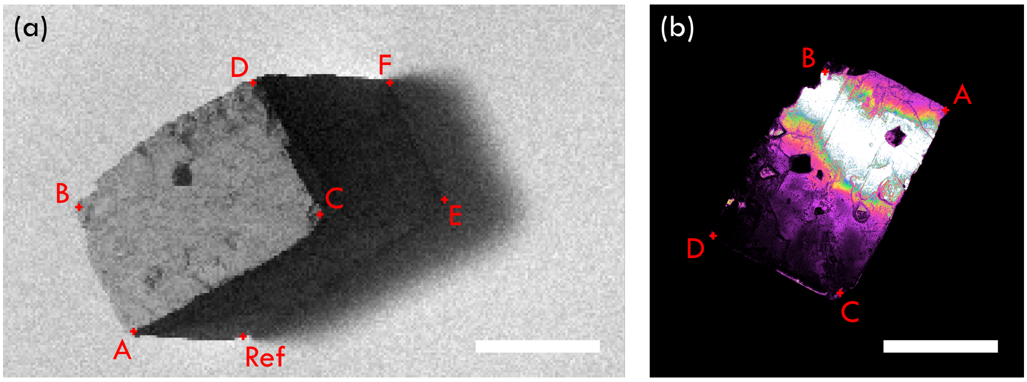

Figure G: Comparative micrographs taken using (a) neutral helium microscopy and (b) confocal laser scanning microscopy. Scale bar 1 mm.

References:

1. Hartley, R. & Zisserman, A. *Multiple view geometry in computer vision*. (Cambridge University Press, 2003).

2. Joseph Goldstein, Dale E. Newbury, David C. Joy, Charles E. Lyman, Patrick Echlin, Eric Lifshin, Linda Sawyer, J. R. M. *Scanning Electron Microscopy and X-ray Microanalysis: Third Edition*. (Springer US, 2003).

3. Fahy, A. *et al.* Image formation in the scanning helium microscope. *Ultramicroscopy* **192,** 7–13 (2018).
